# Deep clinical phenotyping of patients with obsessive-compulsive disorder: an approach towards detection of organic causes and first results

**DOI:** 10.1038/s41398-023-02368-8

**Published:** 2023-03-07

**Authors:** Kimon Runge, Marco Reisert, Bernd Feige, Kathrin Nickel, Horst Urbach, Nils Venhoff, Andreas Tzschach, Miriam A. Schiele, Luciana Hannibal, Harald Prüss, Katharina Domschke, Ludger Tebartz van Elst, Dominique Endres

**Affiliations:** 1 Department of Psychiatry and Psychotherapy, Medical Center - University of Freiburg, Faculty of Medicine, University of Freiburg, Freiburg, Germany; 2 Department of Diagnostic and Interventional Radiology, Medical Physics, Medical Center - University of Freiburg, Faculty of Medicine, University of Freiburg, Freiburg, Germany; 3 Department of Stereotactic and Functional Neurosurgery, Medical Center - University of Freiburg, Faculty of Medicine, University of Freiburg, Freiburg, Germany; 4 Department of Neuroradiology, Medical Center - University of Freiburg, Faculty of Medicine, University of Freiburg, Freiburg, Germany; 5 Department of Rheumatology and Clinical Immunology, Medical Center - University of Freiburg, Faculty of Medicine, University of Freiburg, Freiburg, Germany; 6 Institute of Human Genetics, Medical Center - University of Freiburg, Faculty of Medicine, University of Freiburg, Freiburg, Germany; 7 Laboratory of Clinical Biochemistry and Metabolism, Department of General Pediatrics, Adolescent Medicine and Neonatology, Medical Center - University of Freiburg, Faculty of Medicine, University of Freiburg, Freiburg, Germany; 8 Department of Neurology and Experimental Neurology, Charité - Universitätsmedizin Berlin, Berlin, Germany; 9 German Center for Neurodegenerative Diseases (DZNE), Berlin, Germany; 10 Center for Basics in Neuromodulation, Medical Center - University of Freiburg, Faculty of Medicine, University of Freiburg, Freiburg, Germany

**Keywords:** Diagnostic markers, Molecular neuroscience

## Abstract

In the revised diagnostic classification systems ICD-11 and DSM-5, secondary, organic forms of obsessive-compulsive disorder (OCD) are implemented as specific nosological entities. Therefore, the aim of this study was to clarify whether a comprehensive screening approach, such as the Freiburg-Diagnostic-Protocol for patients with OCD (FDP-OCD), is beneficial for detecting organic OCD forms. The FDP-OCD includes advanced laboratory tests, an expanded magnetic resonance imaging (MRI) protocol, and electroencephalography (EEG) investigations as well as automated MRI and EEG analyses. Cerebrospinal fluid (CSF), [^18^F]fluorodeoxyglucose positron emission tomography, and genetic analysis were added for patients with suspected organic OCD. The diagnostic findings of the first 61 consecutive OCD inpatients (32 female and 29 male; mean age: 32.7 ± 12.05 years) analyzed using our protocol were investigated. A probable organic cause was assumed in five patients (8%), which included three patients with autoimmune OCD (one patient with neurolupus and two with specific novel neuronal antibodies in CSF) and two patients with newly diagnosed genetic syndromes (both with matching MRI alterations). In another five patients (8%), possible organic OCD was detected (three autoimmune cases and two genetic cases). Immunological serum abnormalities were identified in the entire patient group, particularly with high rates of decreased “neurovitamin” levels (suboptimal vitamin D in 75% and folic acid in 21%) and increased streptococcal (in 46%) and antinuclear antibodies (ANAs; in 36%). In summary, the FDP-OCD screening led to the detection of probable or possible organic OCD forms in 16% of the patients with mostly autoimmune forms of OCD. The frequent presence of systemic autoantibodies such as ANAs further support the possible influence of autoimmune processes in subgroups of patients with OCD. Further research is needed to identify the prevalence of organic OCD forms and its treatment options.

## Introduction

Obsessive-compulsive disorder (OCD) is a common and severely disabling mental disorder with a lifetime prevalence of 1 to 3.5% [1–3] and is associated with a significant loss of quality of life [4]. It is assumed that OCD has a multi-factorial pathogenesis with biological, psychological, and social causes, as well as additional external stressors like critical life events or infections [5–7]. Biologically, dysbalance of the cortico–striato–thalamo–cortical circuits, a dysregulation of serotonergic, glutamatergic, or dopaminergic neurotransmissions, as well as (epi-)genetic alterations, are assumed [8–11]. In contrast to these primary forms of OCD, the DSM-5 and the upcoming ICD-11 distinguish obsessive-compulsive symptoms (OCS) in the context of organic causes—i.e., “organic OCD” forms [12, 13] from classical OCD. Organic OCD forms are based on clear-cut organic findings to which OCS can be attributed, like neurological lesions or tumors [14, 15]. In recent years, autoimmune mechanisms have been increasingly discussed in the context of organic OCD [16]. The most prominent example is Pediatric Autoimmune Neuropsychiatric Disorder Associated with Streptococcal Infections (PANDAS) [17–19]. There is also increasing evidence for organic OCD independent of infection due to different neuronal antibodies or in the context of systemic autoimmune disorders such as lupus erythematosus [16, 20–22]. Non-inflammatory causes could include neurodegenerative diseases such as Huntington’s disease or genetic syndromes such as velocardiofacial syndrome (cf. [11]). To date, little is known about the prevalence of organic OCD [9].

### Study rationale

**Fig. 1:**
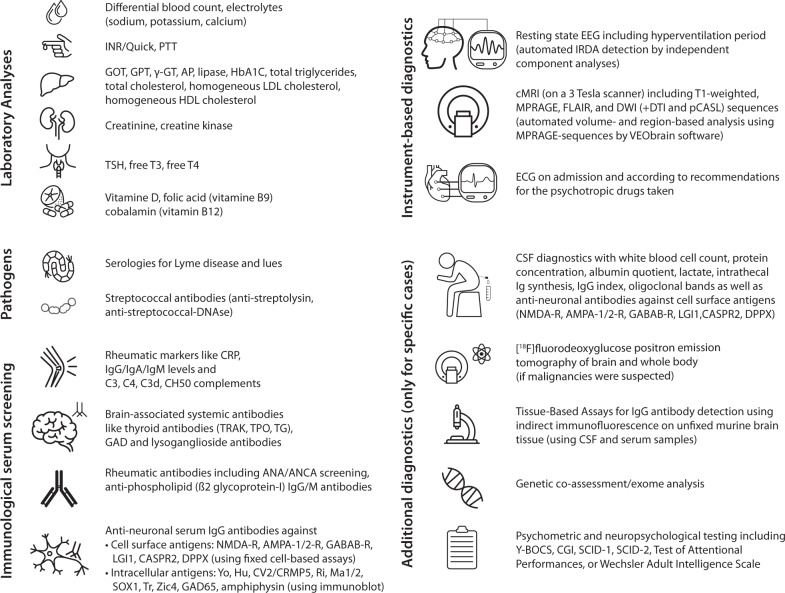
Freiburg diagnostic protocol for patients with OCD (FDP-OCD). AMPA α-amino-3-hydroxy-5-methyl-4-isoxazolepropionic acid, ANA antinuclear antibody, ANCA antineutrophil cytoplasmic antibody, AP alkaline phosphatase, C3/C4/C3d complement component 3/4/3d, CASPR2 Contactin-associated protein-like 2, CGI Clinical Global Impression, CK Creatine kinase, CH50 complement hemolytic activity, CSF Cerebrospinal fluid, cMRI cranial magnetic resonance imaging, CRP C-reactive protein, CRMP5 collapsing response-mediator protein-5, DNAse Deoxyribonuclease, DPPX dipeptidyl-peptidase–like protein 6, DTI Diffusion tensor imaging, DWI Diffusion-weighted imaging, ECG electrocardiogram, EEG Electroencephalogram, FDG-PET fluorodeoxyglucose - positron emission tomography, FLAIR Fluid-attenuated inversion recovery, γ-GT Gamma-glutamyl transferase, GABA Gamma-aminobutyric acid, GAD glutamic acid decarboxylase, GOT Glutamic-Oxaloacetic transaminase, GPT Glutamic-Pyruvic transaminase, HbA1c Hemoglobin A1c, HDL high-density lipoprotein, IgA Immunoglobulin A, IgG Immunoglobulin G, IgM Immunoglobulin M, INR International normalized ratio, LG1 leucine-rich glioma-inactivated 1, LDL low-density lipoprotein, MPRAGE Magnetization Prepared Rapid Acquisition Gradient Echo, NMDA N-methyl-D-aspartate, PCASL pseudo-continuous arterial spin labeling, PTT Partial thromboplastin time, R Receptor, SCID-1/2 Structured Clinical Interview for DSM-IV, T3 Triiodothyronine, T4 Thyroxine, TG Thyroglobulin, TPO Thyroid peroxidase, TRAKs Thyroid-stimulating hormone-receptor-antibodies, TSH Thyroid-stimulating hormone, Y-BOCS Yale-Brown Obsessive Compulsive Scale, Zic4 zinc-finger protein 4.

Organic OCD is gaining clinical importance and has also been included as a diagnosis in the DSM-5 and ICD-11 criteria. However, it is largely unclear how detailed the diagnostic work-up should be for the individual patient. At the Department of Psychiatry and Psychotherapy of the Medical Center, University of Freiburg, Germany, the Freiburg Diagnostic Protocol for patients with OCD (FDP-OCD) was developed (see Fig. 1). This article presents the array of diagnostic abnormalities and number of organic OCD cases when applying the FDP-OCD screening approach in the entire collective of patients with OCD.

## Patients and methods

The FDP-OCD screening approach has been developed over the past years based on the Freiburg Diagnostic Protocol in Psychosis (FDPP) for patients with psychosis [23] and is presented here for the first time together with first results. The retrospective analysis was performed according to the Declaration of Helsinki and after ethical approvement from the Ethics Committee of the University of Freiburg (No. 21 1290).

### Patient group

The first 61 consecutive inpatients (32 females and 29 males with a mean age of 32.7 ± 12.05 years) treated in 2020 and 2021 on our ward specialized for OCD psychotherapy (https://www.uniklinik-freiburg.de/psych/stationen/station-6.html), who agreed to the offered diagnostic work-up, were included in the present study. Patients with the following admission codes according to the criteria of the ICD-10 [24] were analyzed: F42.0 (predominantly obsessional thoughts or ruminations), F42.1 (predominantly compulsive acts), and F42.2 (mixed obsessional thoughts and acts). All diagnoses were assigned based on patient history and clinical examination by experienced senior psychiatrists. Patients under 18, patients with obsessive-compulsive (anankastic) personality disorder (ICD-10: F60.5), and patients with mild cognitive impairment or dementia were excluded. Of the 61 included patients, five suffered from predominantly obsessional thoughts or ruminations (F42.0), six from predominantly compulsive acts (F42.1), and 50 from mixed obsessional thoughts and acts (F42.2).

### FDP-OCD screening

The FDP-OCD screening includes advanced laboratory analysis with screening for pathogens and serum immunological markers, as well as instrumental diagnostics including an extended 3 Tesla magnetic resonance imaging (MRI) protocol, and electrocardiography (ECG). *Extended diagnostic investigations* in patients with suspected organic cases included cerebrospinal fluid (CSF) analysis, [^18^F]fluorodeoxyglucose positron emission tomography (FDG-PET), tissue-based assays on murine brain tissue for the detection of novel anti-central nervous system (CNS) antibodies, genetic and neuropsychological testing (Fig. 1).

#### Laboratory analyses

Blood analyses were offered to all patients as a routine procedure upon admission. The methodology of the tests has been described in previous publications [23, 25–27]. In addition to these routine diagnostic tests, 15 selected patients with suspected autoimmune OCD [16, 28] received a lumbar puncture to investigate an inflammatory cause of OCD. The CSF findings of OCD patients from our hospital were recently summarized in another publication, including patients from other wards and a larger retrospective interval [25, 28]. To discover potential novel anti-CNS antibodies, CSF–serum pairs of 12 patients were investigated on slices of unfixed murine brain tissue by indirect immunofluorescence [29, 30].

#### Instrument-based diagnostics

All patients were offered investigation with EEG (available from *N* = 60), extended MRI sequences of the neurocranium (available from *N* = 57), and ECG (available from *N* = 60). EEGs were examined by the respective ward clinician and retrospectively assessed for regional or focal slowing, intermittent generalized delta/theta activity (IRTA/IRDA), and epileptic activity. Independent component analysis (ICA) of EEGs with automatic calculation of IRDA/IRTA density was additionally performed as previously described [31]. MRI included the following sequences on a 3 Tesla scanner (MAGNETOM Prisma, Siemens Healthcare GmbH, Erlangen, Germany) in most patients: T1-weighted sequences with magnetization-prepared rapid gradient echo (MPRAGE) with isotropic 1-mm^3^ voxels for atrophy diagnostics, diffusion-weighted imaging (DWI) with axial 5-mm slices for stroke detection, fluid-attenuated inversion recovery (FLAIR) sequences with isotropic 1-mm^3^ voxels for the detection of signal alterations, and further innovative analyses such as diffusion tensor imaging (DTI) and pseudo-continuous arterial spin labeling. All MRIs were assessed and evaluated by experienced senior neuroradiologists. MRI abnormalities were categorized as white-/gray-matter alterations, atrophy, vascular changes, cysts, tumors, and anatomical variants, among others. An automated volume- and region-based approach (https://www.VEObrain.com) was used with the MPRAGE sequences for fully automated whole-brain volumetry for the detection of volume loss (VEOmorph, Freiburg, Germany). *Selected cases* (*N* = 7) with abnormalities in routine diagnostic work-up suggestive of an autoimmune cause were examined with cerebral [^18^F]fluorodeoxyglucose positron emission tomography (FDG-PET). Two patients received exome analysis due to suspected syndromal genesis.

#### Sociodemographic and psychometric data

During the admission routine, all important clinical and demographic data were documented. In addition, psychometric scales such as the Global Assessment of Functioning or Clinical Global Impression score were collected. In selected patients, further neuropsychological or psychometric assessments were performed.

### Statistics

The retrospective data were collected and analyzed using SPSS (Version 28; IBM, New York, NY, USA). The analyses were mostly descriptive. The number of pathological values was based on established reference ranges.

## Results

### Sociodemographic and clinical findings

Most patients presented with a chronic course (>2 years) of the disease (78%), with an average of 14.8 years since OCD onset. On average it took six years from onset for OCD to be first diagnosed and patients lived on average 100 km away from the study center in Freiburg, Germany. A large proportion of patients were unmarried (72%), had at least a high school degree (52%), and were employed on a permanent basis (43%). Comorbid mental disorders were observed in nearly three-fourths of patients (74%), with one-quarter presenting with two comorbid diagnoses or more. Depression was by far the most frequent comorbidity (49%). Somatic comorbidities were registered in 82% of participants, with one-quarter of patients having endocrinological disorders. The three most common comorbidities were hypothyroidism (13%), arterial hypertension (8%), and Hashimoto’s thyroiditis (7%). Almost all patients were treated psychotherapeutically according to a cognitive-behavioral therapy protocol entailing exposure and reaction management (97%); in most cases (72%), pharmacological treatment was also provided with antidepressants. All data are summarized in Tables 1 and 2.

**Table 1:** Description of the study cohort.

**Description of the study cohort**			
*Sex*		*Age*	
Female Male	32 (52%) 29 (48%)	Mean ± SD (range), in years	32.67 ± 12.05 (18–69)
*Diagnosis at admission*		*Origin*/*Country of birth*	
F42.2	50 (82%)	Germany	54 (88%)
F42.1	6 (10%)	Europe	4 (7%)
F42.0	5 (8%)	Unknown	3 (5%)
*Duration of illness (OCD)*		*Course of the disease*	
Mean ± SD (range), in years	14.8 ± 11.8 (1–60), (*N* = 51)	Chronic course (>2 years)	48 (78%)
		First episode	6 (10%)
*Distance from home*		Recurrent course	6 (10%)
Mean ± SD (range), in kilometers	92.5 ± 100.4 (1–396), (*N* = 60)	Unknown	1 (2%)
*Inpatient*/*outpatient*		*Time between beginning of illness and diagnosis*	
Inpatient treatment	52 (65%)	Mean ± SD (range), in years	6.0 ± 6.4 (0–30), (*N* = 43)
Part-time inpatient	2 (3%)	*Earlier outpatient psychotherapies*	
Inpatient, then only part-time	7 (12%)	None	10 (16%)
*Earlier inpatient treatments*		One	28 (46%)
None	15 (24%)	Two	16 (26%)
One	15 (24%)	>Two	3 (5%)
Two	8 (13%)	Unknown	4 (6%)
Three	12 (20%)		
Four and more	8 (13%)		
Unknown	3 (5%)		
**Sociodemographic findings of OCD cohort**			
*Civil status*		*Suicide attempts*	
Unmarried	44 (72%)	None	57 (93%)
Married	10 (16%)	One	2 (3%)
Divorced	7 (12%)	Two	2 (3%)
		*School education*	
*Living situation*		Low school degree	5 (8%)
Alone	21 (34%)	Medium school degree	14 (23%)
With partner/family	17 (28%)	High school degree	26 (42%)
With parents/siblings	19 (31%)	University degree	6 (10%)
Psychiatric transitional arrangement	3 (5%)	No degree	2 (3%)
Others/Unknown	1 (2%)	Unknown	8 (14%)
*Occupation*		*Family history for psychiatric diseases*	
Employed	26 (43%)	Positive	37 (61%)
Unemployed	15 (24%)	Negative	17 (28%)
Disability pension	2 (3%)	Unknown	7 (11%)
Retirement pension	2 (3%)		
In-training/in studies/retraining	12 (20%)		
Others	4 (7%)		
*Burden of acute events*		*Burden of long-term life circumstances*	
None	17 (28%)	None	5 (8%)
Mild	8 (13%)	Mild	9 (15%)
Intermediate	17 (28%)	Intermediate	24 (39%)
Severe	15 (24%)	Severe	14 (23%)
Extreme	3 (5%)	Extreme	5 (8%)
Unknown	1 (2%)	Unknown	4 (6%)
**COMORBIDITIES**			
**Mental disorder comorbidity**			
*Number of psychiatric comorbidities*		*Affective disorders*	
Overall	45 (74%)	Depression	30 (49%)
• None	16 (26%)	• mild	5 (8%)
• One	30 (49%)	• moderate	12 (20%)
• Two	8 (13%)	• severe	11 (18%)
• >Two	7 (12%)	• unknown	2 (3%)
*Psychotic disorders*	5 (8%)	Bipolar disorder	0 (0%)
Paranoid hallucinatory	1 (2%)	*Personality disorders*	2 (3%)
Hebephrenic	1 (2%)	Borderline personality disorder	1 (2%)
Schizoaffective	3 (5%)	Avoidant and dependent personality disorder	1 (2%)
*Neurodevelopmental disorders*	7 (11%)		
ADHD	2 (3%)	*Anxiety disorders*	6 (10%)
Autism	5 (8%)	Specific phobia	6 (10%)
Tic disorders	1 (2%)	Generalized anxiety disorder	0 (0%)
*Sleeping disorders*	1 (2%)	Panic disorder	1 (2%)
Narcolepsy with cataplexy	1 (2%)	*Somatoform disorder*	3 (5%)
*Substance abuse*	8 (13%)	*Other disorders*	2 (4%)
Alcohol abuse	6 (10%)	Trichotillomania	1 (2%)
Illegal drug abuse	3 (5%)	Dyslexia	1 (2%)
Benzodiazepine	1 (2%)		
**Somatic comorbidities**			
*Somatic comorbidities*		*Somatic risk factors for psychiatric diseases*	
Overall	50 (82%)	Pregnancy complications	7 (11%)
• None	11 (18%)	Febrile convulsions/seizures	1 (2%)
• One	18 (30%)	Traumatic brain injury	6 (10%)
• Two	12 (20%)	Previous meningitis/encephalitis	0 (0%)
• Three	10 (16%)	Severe infectious diseases	1 (2%)
• Four and more	10 (16%)		
**Neurological comorbidities**			
Neurological overall	6 (10%)	*Headache (e.g., migraine)*	
Neuropsychiatric lupus erythematosus^a^	1 (2%)	• Migraine without aura	1 (2%)
*Neurovascular*		• Migraine not specified	1 (2%)
Cerebral microangiopathy	2 (3%)	Circulation (e.g., syncope)	1 (2%)
Progressive subcortical vascular encephalopathy	1 (2%)	Traumatic injuries	1 (2%)
Epilepsy (structural epilepsy)	1 (2%)	*Others*	
		Colloid cyst	1 (2%)
		Intracerebral cavernoma	1 (2%)
**Internistic comorbidity**			
*Cardiological*	11 (18%)	*Hematological*	4 (7%)
Arterial hypertension	5 (8%)	Morbus Werlhof	1 (2%)
Chronic venous insufficiency	1 (2%)	Pseudothrombocytopenia	1 (2%)
Aortic root dilation	1 (2%)	Agranulocytosis	1 (2%)
AV nodal reentry tachycardia	1 (2%)	*Nephrological*	
Aortic valve sclerosis with mild to moderate aortic valve regurgitation	1 (2%)	Chronic kidney disease stage 3	1 (2%)
Decent carotid bulb plaque	1 (2%)	*Endocrinological*	15 (25%)
Open foramen ovale	1 (2%)	Hypothyroidism	8 (13%)
*Malignancies*/*tumors*	5 (8%)	Hashimoto’s thyroiditis	4 (7%)
Diffuse large B-cell lymphoma	1 (2%)	Hypertriglyceridaemia	2 (3%)
Testicular carcinoma	1 (2%)	Hyperprolactinaemia	2 (3%)
Haemangioma in the hepatobilliary system and pancreas	1 (2%)	Diabetes mellitus type 2	1 (2%)
Pituitary macroadenoma	1 (2%)	*Infectiological*	9 (15%)
Colon polyps	1 (2%)	Dermatological	3 (5%)
*Gastroenterological*	7 (12%)	Infectious mononucleosis with EBV	1 (2%)
Irritable Bowel Syndrome	2 (3%)	Ophthalmic zoster	2 (3%)
Gastroesophageal reflux disease	2 (3%)	History of Lyme disease	3 (5%)
Abdominal vasosclerosis	1 (2%)	*Pneumological*	5 (8%)
Others (gastritis, appendicitis, hemorrhoids)	3 (5%)	Allergy/allergic asthma	4 (7%)
*Rheumatologic/autoimmune*	4 (7%)	Obstructive sleep apnea syndrome	1 (2%)
Neurolupus	1 (2%)		
Spondylarthritis with HLA-B27 positivity	1 (2%)		
Fibromyalgia syndrome	1 (2%)		
Celiac disease	1 (2%)		
**Other somatic comorbidities**			
*Urogenital diseases*	5 (8%)	*Gynaecological diseases*	3 (5%)
Urogenital infections (cystitis, urethritis)	2 (4%)	Polycystic ovarian syndrome	1 (2%)
Hypospadias	1 (2%)	Premature ovarian failure	1 (2%)
Varicocele	1 (2%)	Pregnancy, puerperium, birth	1 (2%)
Others (incontinence, narrow meatus)	2 (4%)	*Dermatologic diseases*	7 (12%)
*Otorhinolaryngologic diseases*	3 (5%)	Eczema	2 (4%)
Tinnitus aurium	1 (2%)	Others (acne, furuncle, ictus reaction)	5 (8%)
Hearing loss	2 (4%)	*Genetic syndromes*	4 (7%)
*Muscle, skeleton and connective tissues disorders*	7 (12%)	Mayer–Rokitansky–Kuster–Hauser syndrome^a^	1 (2%)
Tendopathy	3 (5%)	Duplication of chromosome 15q11-13^a,b^	1 (2%)
Musculoskeletal injury	3 (5%)	Turner syndrome	1 (2%)
Drug-induced rhabdomyolysis	1 (2%)	Triple-X syndrome	1 (2%)
Coxarthrosis	1 (2%)	Baraitser–Winter syndrome^b^	1 (2%)

Some parameters were not available from all patients, then the reduced sample size is always indicated in parentheses. *ADHD* Attention deficit hyperactivity disorder, *AV* atrioventricular, *EBV* Epstein-Barr virus, *HLA* human leukocyte antigen, *N* number of patients, *OCD* obsessive compulsive disorder, *SD* standard deviation.

^a^The same patient suffered from both syndromes.

^b^Newly diagnosed.

**Table 2:** Psychometric findings and therapy.

**Psychometric findings and therapy**			
*GAF on admission*		*CGI on admission*	
Mean ± SD (range)	48.4 ± 13.3 (22–81), (*N* = 61)	Borderline ill	0 (0%)
		Mildly ill	0 (0%)
		Moderately ill	1 (2%)
		Markedly ill	15 (25%)
		Severely ill	38 (62%)
		Extreme severely ill	5 (8%)
		Unknown	2 (3%)
**Treatment of inpatients**			
*Overall psychopharmacological treatment on admission*		*Number of psychopharmaceutical classes per patient*	
Psychopharmacological treatment received	44 (72%)	No treatment	17 (28%)
No psychopharmacological treatment received	17 (28%)	One class	27 (44%)
*Psychopharmacological treatment on admission in detail*		Two classes	15 (25%)
SSRI	32 (53%)	Three classes	2 (3%)
Atypical neuroleptics	13 (21%)	*Psychotherapy concept treatment on the OCD ward overall*	
Typical neuroleptics	4 (7%)	No	2 (3%)
Lithium	2 (3%)	Yes	59 (97%)
Anticonvulsants	2 (3%)		
Benzodiazepines	2 (3%)		
Stimulants	2 (3%)		
Clonidine	1 (2%)		
Clomipramine	3 (5%)		
Other antidepressants	13 (21%)		

*CGI* clinical global impression, *GAF* global assessment of functioning, *SD* standard deviation, *N* numbers of patients, *OCD* obsessive compulsive disorder, *SSRI* Selective serotonin reuptake inhibitors.

### Laboratory blood findings

In serum analysis, reduced leukocyte counts were identified in 10% of patients with OCD. Furthermore, increased calcium levels were found in 18% of participants, partial thromboplastin times (PTT) were decreased in 14%, GPT was increased in 12%, and increased total glycerides were detectable in 25% of the patients. Regarding vitamins, OCD patients presented with decreased vitamin D levels in 35% of cases—up to 75% when applying the optimal value of 30 ng/ml—and a folic acid deficiency was found in 24%. Signs for current Lyme disease in serology were identified in 6% of patients, whereby evidence of past Lyme disease was identified in 7%. Streptococcal antibodies were detected in 46% of the patients (anti-streptolysin in 38%, streptococcal-DNAseB in 23%). Rheumatological screening revealed aberrant rheumatic markers (CRP, immunoglobulins, complement system) in 34%, with elevated CRP levels in 16%. Furthermore, positive rheumatic serum autoantibodies were observed in 42% of patients, with 36% presenting with positive antinuclear antibodies (ANA) tested by indirect immunofluorescence (IIF) on human epithelial type 2 (HEp-2) cells (16% clearly positive and 20% borderline) and 10% with elevated anti-phospholipid antibodies. In addition, positive anti-thyroid antibodies were identified in 24%, with positive antibody findings for anti-thyroid peroxidase (TPO) in 19% and anti-thyroglobulin (TG) in 13%, but none against the thyroid-stimulating hormone receptor.

**Fig. 2:**
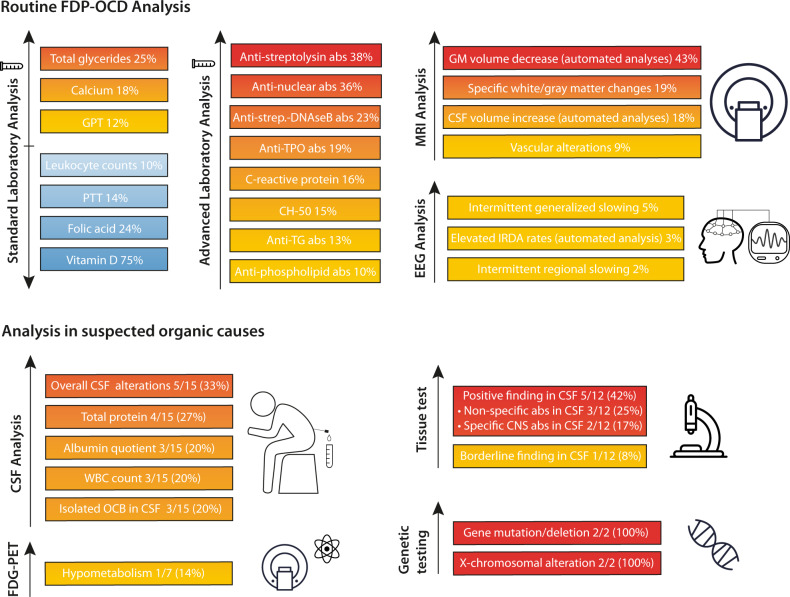
Prevalence of laboratory, MRI, and EEG alterations in OCD patients. Elevated parameters are displayed in yellow/orange/red depending on level of elevation. Decreased parameters are displayd in blue tones. CH50 complement hemolytic activity, CSF Cerebrospinal fluid, DNAse Deoxyribonuclease, EEG Electroencephalogram, GM gray matter, MRI magnetic resonance imaging, OCB oligoclonal band, PTT Partial thromboplastin time, TG Thyroglobulin, TPO Thyroid peroxidase, WBC white blood cell.

Well-characterized anti-neuronal serum antibodies against cell-surface (NMDA-R/LGI1/CASPR2/AMPA1/2-R/GABA-B-R/DPPX) and intracellular antigens (Yo/Hu/CV2[CRMP5]/Ri/Ma1/2/SOX1/Tr/Zic4/GAD65/amphiphysin) were negative in all OCD patients. Tissue-based assays on murine brain slices detected abnormal serum findings in five of 13 (38%) patients examined. This included one finding with binding against neuropil in the basal ganglia and brain stem [20] and one with a novel cytoplasmic pattern [32]. The other three patients had strong antinuclear binding with a perinuclear pattern. In addition, in two patients, a borderline finding with an anti-myelin-binding pattern was observed. All laboratory results are summarized in Table 3 and prevalences of laboratory, MRI and EEG alterations in OCD are presented in Fig. 2.

**Table 3:** Laboratory findings.

STANDARD LABORATORY ANALYSIS		
Parameter (reference values)	Mean ± Standard deviation	Patients with increased/decreased values
***Differential blood count***		
Leukocytes (4000–1040/µl)	6330 ± 2000/µl (*N* = 61)	***3*** ↑ ***(5%), 6*** ↓ ***(10%), 52 normal (85%)***
Platelets (150,000–400,000/µl)	241,980 ± 55,340/µl (*N* = 61)	4 ↓ (7%), 57 normal (93%)
Erythrocytes (♂: 4.5–5.8 mio/µl, ♀: 4.0–5.2 mio/µl)	4.80 ± 0.47 mio/µl (*N* = 61)	2 ↓ (7%), 59 normal (93%)
MCV (80.0–95.5 fl)	87.57 ± 4.30 fl (*N* = 61)	2 ↑ (3%), 1 ↓ (2%), 58 normal (95%)
MCH (27.6–32.8 pg)	30.15 ± 1.46 pg (*N* = 61)	4 ↑ (7%), 57 normal (93%)
MCHC (32.8–36.6 g/dl)	34.45 ± 0.94 g/dl (*N* = 61)	**9** ↑ **(15%), 52 normal (85%)**
Hemoglobin (♂: 13.5–17.6 g/dl, ♀: 11.6–15.5 g/dl)	14.47 ± 1.38 g/dl (*N* = 61)	61 normal (100%)
Hematocrit (34.6–45.3%)	41.97 ± 3.57% (*N* = 61)	61 normal (100%)
Neutrophil granulocytes (40–75%)	55.31 ± 10.05% (*N* = 59)	1 ↑ (2%), 3 ↓ (5%), 55 normal (93%)
Lymphocytes (19–51%)	32.55 ± 8.70% (*N* = 59)	1 ↑ (2%), 3 ↓ (5%), 55 normal (93%)
Eosinophils (0–7%)	2.43 ± 1.58% (*N* = 59)	1 ↑ (2%), 58 normal (98%)
Basophile (0–2%)	0.69 ± 0.33% (*N* = 59)	59 normal (100%)
Monocytes (2–14%)	8.77 ± 2.55% (*N* = 59)	1 ↑ (2%), 58 normal (98%)
Neutrophil absol. (1.8–6.2 tsd/µl)	3550 ± 1470 tsd/µl (*N* = 59)	4 ↑ (7%), 3 ↓ (5%), 52 normal (88%)
Lymphocytes absol. (1200–3600/µl)	1920 ± 520/µl (*N* = 59)	5 ↓ (9%), 54 normal (91%)
Eosinophile absol. (30–440/µl)	150 ± 110/µl (*N* = 59)	2 ↓ (3%), 57 normal (97%)
Basophile absol. (10–80/µl)	40 ± 20/µl (*N* = 59)	59 normal (100%)
Monocytes absol. (250–850/µl)	530 ± 170/µl (*N* = 59)	1 ↑ (2%), 58 normal (98%)
***Coagulation***		
Quick (70–130%)	98.77 ± 12.70% (*N* = 56)	56 normal (100%)
INR (0.85–1.15)	1.02 ± 0.05 (*N* = 58)	58 normal (100%)
PTT (25.1–37.7 s)	29.38 ± 3.79 sec (*N* = 58)	**2** ↑ **(3%), 8** ↓ **(14%), 48 normal (83%)**
***Electrolytes***		
Sodium (136–145 mmol/l)	139.85 ± 1.64 mmol/l (*N* = 61)	1 ↓ (2%), 60 normal (98%)
Potassium (3.5–5.1 mmol/l)	4.26 ± 0.30 mmol/l (*N* = 61)	1 ↓ (2%), 60 normal (98%)
Calcium (2.15–2.5 mmol/l)	2.41 ± 0.10 mmol/l (*N* = 61)	**11** ↑ **(18%), 49 normal (82%)**
***Metabolic markers***		
Creatinine (0.51–0.95 mg/dl)	0.86 ± 0.17 mg/dl (*N* = 61)	2 ↑ (3%), 1 ↓ (2%), 58 normal (95%)
CK (<170 U/l)	122.35 ± 126.273 U/l (*N* = 55)	5 ↑ (9%), 50 normal (91%)
GOT (10–35 U/l)	23.95 ± 7.55 U/l (*N* = 60)	4 ↑ (7%), 56 normal (93%)
GPT (10–35 U/l)	23.64 ± 11.19 U/l (*N* = 61)	***7*** ↑ ***(12%), 2*** ↓ ***(3%), 52 normal (85%)***
Alkaline phosphatase (♂: 40–130 U/l, ♀: 35–105 U/l)	72.15 ± 21.77 U/l (*N* = 54)	2 ↑ (4%), 52 normal (96%)
Gamma-GT (<40 U/l)	23.15 ± 24.70 U/l (*N* = 61)	5 ↑ (8%), 56 normal (92%)
Lipase (13–60 U/l)	33.07 ± 16.42 U/l (*N* = 57)	2 ↑ (3%), 55 normal (97%)
HbA1C (3.4–6%)	5.29 ± 0.29% (*N* = 54)	1 ↑ (2%), 53 normal (98%)
Total triglycerides (<150 mg/dl)	112.84 ± 58.59 mg/dl (*N* = 55)	**14** ↑ **(25%), 41 normal (75%)**
***Thyroid hormones***		
TSH (0.27–4.20 µU/ml)	1.80 ± 0.82 µU/ml (*N* = 60)	2 ↓ (3%), 58 normal (97%)
Free T3 (3.1–6.8 pmol/l)	5.14 ± 0.75 pmol/l (*N* = 58)	58 normal (100%)
Free T4 (12–22 pmol/l)	15.51 ± 2.59 pmol/l (*N* = 58)	4 ↓ (7%), 54 normal (93%)
***“Neurovitamins”***		
25-OH-Vitamin D2/D3 (>20 ng/ml)	24.48 ± 10.58 ng/ml (*N* = 57)	**20** ↓ **(35%), 37 normal (65%)**
•Optimal 25-OH-Vitamin D2/D3 (>30 ng/ml)		**43** ↓ **(75%), 14 normal (25%)**
Folic acid (4.8–37.3 ng/ml)	7.73 ± 4.66 ng/ml (*N* = 54)	**13** ↓ **(24%), 41 normal (76%)**
Vitamin B12 (197–771 pg/ml)	432.36 ± 179.78 pg/ml (*N* = 55)	2 ↑ (3%), 1 ↓ (2%), 52 normal (95%)

ADVANCED LABORATORY ANALYSIS		
Parameter (Reference)	Patients with positive/negative/borderline findings or increased/decreased values	
***Pathogen findings***		
*Serology for Lyme disease*		
IgG-ELISA screening (up to 16 U)	***7 positive (13%), 46 negative (83%), 2 bord. (4%) (N*** = ***52)***	
IgM-ELISA screening (up to 5 U)	***3 positive (6%), 43 negative (83%), 6 bord. (11%) (N*** = ***52)***	
Western blot confirmation test (only performed if ELISA screening was conspicuous)	- IgG: positive: 4 (7%), bord.: 1 (2%), negative: 4 (7%) - IgM: positive: 3 (6%), bord.: 1 (2%), negative: 5 (10%) - Both IgG and IgM positive: 2 (4%)	
*Serology for lues*		
Screening using Chemiluminescence Immunoassays (<0.9)	52 normal (100%), (*N* = 52)	
*Streptococcal antibodies*		
Anti-streptolysin (≥200 IU/ml positive)	**18** ↑ **(38%), 27 normal (60%)**	
	Mean ± SD: 190.04 ± 131.84 (*N* = 48)	
Anti-streptococcal-DNAseB U/ml (≥300 positive)	**11** ↑ **(23%), 36 normal (77%)**	
	Mean ± SD: 240.58 ± 322.22 (*N* = 47)	
***Immunological findings***		
*Rheumatic/immunological serum markers*		
C-reactive protein (<5 mg/l)	**9** ↑ **(16%), 48 normal (84%) (*N*** = **57)**	
IgG (7–16 g/l)	3 ↓ (6%), 52 normal (94%) (*N* = 55)	
	Mean ± SD: 11.03 ± 2.14 (*N* = 55)	
IgA (0.70–4 g/l)	2 ↑ (4%), 3 ↓ (5%), 50 normal (91%) (*N* = 57)	
	Mean ± SD: 2.05 ± 0.86 (*N* = 55)	
IgM (0.40–2.30 g/l)	1 ↑ (2%), 4 ↓ (7%), 52 normal (91%) (*N* = 57)	
	Mean ± SD: 0.99 ± 0.50 (*N* = 55)	
C3 (0.90–1.80 g/l)	1 ↑ (2%), 3 ↓ (5%), 51 normal (93%) (*N* = 55)	
C4 (0.10-0.40 g/l)	55 normal (100%) (*N* = 55)	
CH50 (65–115%)	**8** ↑ **(15%), 1** ↓ **(2%), 44 normal (83%) (*N*** = **53)**	
**Rheumatic markers overall**	**20 positive (34%), 38 negative (66%) (*N*** = **58)**	
*Brain-associated systemic antibodies*		
Anti-TSHR antibodies (<1.75 IU/l)	54 normal (100%) (*N* = 54)	
Anti-TPO antibodies (<34 IU/ml)	**10** ↑ **(19%), 44 normal (81%) (*N*** = **54)**	
Anti-TG antibodies (<115 IU/ml)	***7*** ↑ ***(13%), 47 normal (87%) (N*** = ***54)***	
Anti-GAD65 antibodies measured by RIA (< 1 U/ml, bord. 1–2 U/ml, positive >2 U/ml)	1 positive (2%), 1 bord. (2%), 50 normal (96%) (*N* = 52)	
Lysoganglioside antibodies (GM1 IgG <10 normal, bord.: <15, positive >15)	35 normal (100%) (*N* = 35)	
**Anti-thyroid and diabetes antibodies overall**	**13 positive (24%), 41 negative (76%) (*N*** = **54)**	
*Rheumatic serum autoantibodies*		
Anti-phospholipid/ß2GP IgG antibodies (<14 GPL-U/ml)	3 ↑ (6%), 50 normal (94%) (*N* = 53)	
Anti-phospholipid /ß2GP IgM antibodies (<10 MPL-U/ml)	2 ↑ (4%), 52 normal (96%) (*N* = 53)	
ß2Glycoprotein1AKIgG	Positive 1 (2%), negative 50 (98%) (*N* = 51)	
ANA screening by indirect immunofluorescence (ANA-IIF) using Human Epithelial type 2 (Hep-2) cells		
• against nucleus (1:50)	**Positive 3**^**a**^ **(5%), bord. 14 (25%), negative 38 (69%) (*N*** = **55)**	
• against nucleoli (1:50)	Positive 1 (2%), negative 54 (98%) (*N* = 55)	
• against chromosomes (1:50)	***Positive 7***^a^ ***(13%), bord. 7 (13%), negative 41 (75%) (N*** = ***55)***	
• against cytoplasm (1:50)	Bord. 4 (7%), negative 51 (93%) (*N* = 55)	
ANA overall positive/borderline findings	**Positive 9 (16%), bord. 11 (20%), negative 35 (64%) (*N*** = **55)**	
ENA-Screening (only performed if ANA-IIF screening was clearly positive)	Positive 4 (7%), negative 6 (11%) (*N* = 10) Positive cases in detail: One patient with anti-Jo1 and anti-mitochondrial M2 abs as well as two patients with anti PCNA (proliferating cell nuclear antigen) abs. One patient had repeated anti-nucleosome abs. in the past due to lupus. One patient with anti DFS-70 abs.	
Anti-dsDNA ELISA ( < 40 U/ml; only performed if ANA-IIF screening was clearly positive and suspicious clinical findings were present)	Negative 7 (100%) (*N* = 7)	
ANCA (IgG, 1:10)	Positive 0 (0%), negative 57 (100%) (*N* = 57)	
**Rheumatic autoantibodies overall**	**Positive 23 (42%), negative 32 (58%) (*N*** = **55)**	
*Well-characterized neuronal serum autoantibodies*		
Antibodies against intracellular onconeural antigens (*Hu, Yo, Ri, cv2(CRMP5), Ma1/ -Ma2, SOX, Tr(DNER), Zic4, amphiphysin, GAD65*)	(*N* = 52) Positive 0 (0%) Negative 52 (100%)	
Antibodies against neuronal cell surface antigens *(LGI1, CASPR2, GABA-B, NMDA-R, AMPA 1/2*)	(*N* = 51) Positive 0 (0%) Negative 51 (100%)	
*Tissue-based assays (Prof. Prüss, Charité Berlin, Germany) in serum*		
	(*N* = 13)	
None/weak/unspecific (negative)	6/13 (46%)	
Moderate/specific (borderline)	2/13 (15%)	
Strong/specific (positive)	5/13 (38%)	

All results, which were altered for more than 10 percent of patients are in bold and italic, and with more than 15 percent only in bold.

↓ decreased, ↑ increased, *abs* antibodies, *absol.* absolute, *ANA* antinuclear antibodies, *ANCA* antineutrophil cytoplasmic antibody, *bord.* borderline, *bord.* borderline, *C3* complement component 3, *C4* complement component 4, *CASPR2* Contactin-associated protein-like 2, *CH50* 50% hemolytic complement, *CK* creatine kinase, *CRMP5* collapsing response-mediator protein-5, *DFS* dense fine speckled, *DNA* deoxyribonucleic acid, *DNER* delta/notch-like epidermal growth factor-related receptor, *dsDNA* double stranded deoxyribonucleic acid, *ELISA* Enzyme-linked immunosorbent assay, *ELISA* enzyme-linked immunosorbent assay, *ENA* extractable nuclear antigens, *GABA-B* gamma-aminobutyric acid receptor B, *GAD* glutamic acid decarboxylase, *Gamma-GT* Gamma-glutamyl transferase, *GOT* glutamic-oxaloacetic transaminase, *GPT* Glutamic-Pyruvic transaminase, *HbA1c* hemoglobin A1c, *IgA* immunoglobulin A, *IgG* immunoglobulin G, *IgG* immunoglobulin G, *IgM* immunoglobulin M, *IgM* immunoglobulin M, *INR* international normalized ratio, *LG1* Leucine-rich glioma-inactivated 1, *MCH* Mean corpuscular hemoglobin, *MCHC* mean corpuscular hemoglobin concentration, *MCV* Mean corpuscular volume, *MPA* α-amino-3-hydroxy-5-methyl-4-isoxazolepropionic acid, *N* numbers of patients, *NMDA* N-methyl-D-aspartate, *PCNA* proliferating cell nuclear antigen, *PTT* partial thromboplastin time, *RIA* radioimmunoassay, *SD* standard deviation, *ß2GP* ß2 glycoprotein, *T3* triiodothyronine, *T4* thyroxine, *TG* thyroglobulin, *TPO* thyroid peroxidase, *TSH* thyroid-stimulating hormone, *TSHR* thyroid-stimulating hormone-receptor, *Zic4* zinc-finger protein 4.

^a^Including one patient with lupus and highly positive ANA-titers with repeated anti-nucleosomes abs. in the past before treatment.

### Magnetic resonance imaging

#### Clinical assessment

MRI revealed abnormalities such as cerebral lesions, anatomic abnormalities, cysts, or tumors in 15 patients (26%). However, only 11 findings (19%) were rated as potentially relevant for mental disorders with specific gray or white matter changes. These findings included lesions in the right precentral gyrus, left tapetum, left thalamus, trigonum of a ventricle or other potential post-inflammatory localizations cortical, juxtacortical, and periventricular. No relevant atrophy was reported on visual assessment (Table 4).

**Table 4:** Magnetic resonance imaging (MRI) findings in clinical assessment and using automated Veobrain analysis (available in most patients), as well as electroencephalography (EEG) results.

Clinical MRI assessment (*N* = 57)	Number of patients with changes
*White/gray matter changes*	
Specific changes vs no specific changes	11 (19%) vs. 46 (81%)
Non-specific white matter changes	12 (21%)
(Post-)inflammatory white matter changes	6 (11%)
Parenchymal defect	2 (4%)
*Atrophic changes*	
No atrophy	57 (100%)
Generalized/localized cortical atrophy	0 (0%)
Ventricle enlargement	0 (0%)
*Vascular changes*	
Macroangiopathic vascular alterations (post-ischemic changes)	2 (4%)
Microhaemorrhage	3 (5%)
*Cysts, tumors, anatomical variants & other changes*	
Cysts	
• Pineal cyst	3 (5%)
• Arachnoid cyst	2 (4%)
• Pars intermedia cyst	1 (2%)
Tumors	
• Meningioma	1 (2%)
Anatomical variants	
• Partial empty sella	1 (2%)
• Virschow Robin spaces in loco typico	1 (2%)
*Overall MRI changes*	
• Relevant abnormalities	11 (19%)
• Any changes (incl. non-specific changes)	15 (26%)
• No changes	42 (74%)

Automated Veobrain analyses (*N* = 44)	Number of patients with changes	Mean ± SD percentage of voxel with z-score <−2 for gray matter decrease or >3 for CSF volume increase (only patients with changes)
*CSF volume increase*		
Orbitofrontal	2 (5%)	10.22 ± 0.81
	(R:1/L:2)	(R:10.79 /L:9.46 ± 0.28)
Frontal	1 (2%); (R:0/L:1)	8.74
Parietal	1 (2%) (R:1/L:0)	7.28
Temporal	0 (0%)	
Mesiotemporal	4 (9%)	7.37 ± 2.88
	(R:3/L:3)	(R:8.43 ± 2.37/L:6.50 ± 2.03)
Occipital	3 (7%)	7.16 ± 2.43
	(R:3/L:3)	(R:6.84 ± 3.34/L:7.56 ± 0.34)
Cerebellum	1 (2%)	5.73
Overall	8 (18%)	
*Gray matter volume decrease*		
Orbitofrontal	7 (16%)	13.92 ± 7.82
	(R:6/L:6)	(R: 15.54 ± 7.18/L:11.13 ± 6.66)
Frontal	7 (16%)	8.33 ± 3.27
	(R:7/L:4)	(R:7.81 ± 2.50/L:8.16 ± 4.24)
Parietal	7 (16%)	9.21 ± 3.06
	(R:4/L:6)	(R:9.10 ± 3.47/L:9.49 ± 2.57)
Temporal	6 (14%)	7.55 ± 2.72
	(R:2/L:5)	(R: 9.44 ± 0.26/L:7.13 ± 2.82)
Mesiotemporal	3 (7%)	18.36 ± 10.64
	(R:2/L:2)	(R:13.06 ± 7.57/L:20.93 ± 1139)
Occipital	8 (18%)	8.54 ± 3.39
	(R:7/L:6)	(R:7.83 ± 3.57/L:9.24 ± 3.49)
Central	1 (2%) (R:0/L:1)	10.75
Cerebellum	1 (2%)	10.06
Overall	19 (43%)	
*Overall changes in Veobrain analyses*	20/44 (45%)	

EEG findings (*N* = 60)	
**Alterations**	Number of patients with changes
Overall alterations	4 (7%)
No alterations	56 (93%)
Continuous generalized/reginal slow activity	0 (0%)
Intermittent generalized slow activity	3 (5%)
Intermittent regional slow activity	1 (2%)
Epileptic pattern	0 (0%)
Other alterations (e.g., anteriorization of the basic activity)	0 (0%)
**Automated IRDA/IRTA detection**	Mean ± SD (range)
IRDA/IRTA rate before hyperventilation (per min.)	1.12 ± 2.46 (0.00–17.30), (*N* = 60)
IRDA/IRTA rate after hyperventilation (per min.)	1.26 ± 2.83 (0.00–18.82), (*N* = 53)
Difference in IRDA/IRTA rates before and after hyperventilation (per min.)	0.69 ± 0.82 (0.00–3.2), (*N* = 53)
IRDA/IRTA rate overall (per min.)	1.18 ± 2.51 (0.00–18.04), (*N* = 60)
Clinically relevant IRDA/IRTA-rate of more than 6 per minute	*N* = 2 (3%)

*CSF* cerebrospinal fluid, *EEG* electroencephalography, *IRDA/IRTA* intermittent rhythmic delta and theta activity, *L* left, *MRI* magnetic resonance imaging, *N* number of patients, *R* right, *SD* standard deviation.

#### Automated MRI analysis

Fully automated analyses of 44 patients (only possible for patients with available MPRAGE sequence) showed alterations in 20 (45%) of them. Most prominently volume reduction, with a mean percentage of voxel with z-score < −2, of occipital (18%), but also orbitofrontal, frontal and parietal (each 16%) cortex were observed. A CSF volume increase, with percentage of voxel with z-score > 3, was detected mostly in mesiotemporal areas (9%). If affected, the mesiotemporal cortex with 18.36 and the orbitofrontal cortex with 13.92 deviated the most from the reference group. All four patients with genetic syndromes and OCD had orbitofrontal abnormalities.

### EEG findings

EEG revealed changes in visual assessment in the form of slowing in 7% of all patients (Table 4). In the automatic IRDA/IRTA detection, two patients (3%) were observed with increased IRDA/IRTA rates (in-house ref.: rate of more than 6 per minute). These were already visually classified with mainly regional slow activity. The ECG findings are summarized in Supplementary Table S1.

### Further analyses in selected cases

For CSF analysis, five of the 15 patients who underwent lumbar puncture presented abnormalities in the form of elevated WBC count (*N* = 3), elevated protein concentrations (*N* = 4), elevated IgG index (*N* = 1), and oligoclonal bands (OCBs) in CSF (*N* = 3). Of the 12 patients in which tissue-based assays were performed using CSF, five patients presented clearly positive antibody patterns. Two had specific neuronal antibody patterns—one patient each with a pattern against neuropil in basal ganglia and brain stem [33] and one patient with antibodies against the cilia of granule cells [34]. Three patients showed non-specific findings with strong antinuclear binding with partly perinuclear patterns in two of them. In addition, there was a borderline finding for one patient with an anti-myelin-binding pattern.

In the FDG-PET analysis of seven patients, only one showed alterations (see below; Table 5). Two patients were clinically suspected of having a previously undiagnosed genetic syndrome. An exome analysis was conspicuous in both patients (see below).

**Table 5:** Additional analyses in a subgroup of patients.

Cerebral spinal fluid (*N* = 15) and FDG-PET analysis (*N* = 7)	
*WBC count* *(Mean* *±* *SD, range)*	3.20 ± 3.91 (from 1 to 14/µl)
*Increased WBC count*^*a*^ (ref. <5/µl)	↑: 3^a^ (20%) ↔: 12 (80%)
*Protein concentration (Mean* *±* *SD, range)*	380.40 ± 193.86 (from 159 to 927 mg/l)
*Increased total protein concentration* (ref. <450 mg/l)	↑: 4 (27%)
	↔: 11 (73%)
*Albumin quotients (Mean* *±* *SD, range)*	5.42 ± 3.06 (from 2.90 to 14.30 × 10^−3^)
*Increased albumin quotients* (ref.: <40y.: <6.5 × 10^−3^; 40–60y.: <8 × 10^−3^; >60y.: <9.3 × 10^−3^)	↑: 3 (20%)
	↔: 12 (80%)
*IgG-Index (Mean* *±* *SD, range)*	0.54 ± 0.23 (from 0.41 to 1.37)
*Number of patients with increased IgG indices* (ref. <0.7)	↑: 1 (7%)
	↔: 14 (93%)
*Isolated OCB in CSF*	3/15 (20%)
*OCBs in CSF and Serum*	0/15 (0%)
*Overall basic CSF alterations*	5 (33%) (*N* = 15)
*Neuronal CSF antibodies*	
Antibodies against neuronal cell surface antigens (*LGI1, CASPR2, GABA-B, NMDA-R, AMPA 1/2*)	Positive 0 (0%) (*N* = 15)
	Negative 15 (100%) (*N* = 15)
*Tissue based assays (Prof. Prüss, Charité Berlin, Germany)*	(*N* = 12)
None/weak/unspecific (negative)	6/12 (50%)
Moderate/specific (borderline)	1/12 (8%)
Strong/specific (positive)	5/12 (42%)
*FDG-PET*	
Normal	6 (86%)
Hypermetabolism	0 (0%)
Hypometabolism	1 (14%)

*AMPA* α-amino-3-hydroxy-5-methyl-4-isoxazolepropionic acid, *CASPR2* Contactin-associated protein-like 2, *CSF* Cerebrospinal fluid, *FDG-PET* fluorodeoxyglucose - positron emission tomography, *GABA-B* gamma-aminobutyric acid receptor B, *IgG* Immunoglobulin G, *LG1* Leucine-rich glioma-inactivated 1, *n/N* numbers of patients, *NMDA* N-methyl-D-aspartate, *OCBs* Oligoclonal bands, *ref.* reference, *SD* standard deviation, *WBC* white blood cell, ↑ increased, ↓ decreased, ↔ no change.

^a^Including one patient with neurolupus with pleocytosis and positive OCBs in the CSF in the past.

### Organic OCD cases

Probable organic OCD was detectable in a total of five patients (8%) and possible organic OCD was detected in another five patients (8%).

#### Probable autoimmune OCD

The following autoimmune OCD cases were identified as probable organic froms: A female patient with pleocytosis, granule cell and endothelial cell antibodies as well as a reversible MRI lesion at the tapetum [34] (Patient 1), a patient with antibodies against basal ganglia structures in the CSF, autoimmune susceptibility (different autoantibodies in serum), and MRI changes with bilateral small white-matter lesions and a paraventricular defect along the left posterior horn, as well as a bilateral orbitofrontal volume reduction in automated MRI analyses [20] (Patient 2), and another autoimmune patient had pre-diagnosed neuropsychiatric lupus with inflammatory CSF changes and periventricular white-matter lesions (Patient 3). All three patients showed relevant improvement after initial high-dose steroid treatment over five days (in patient 1 decrease of Y-BOCS-scores from 29 to 0 after approx. 7 weeks; in patient 2 decrease of Y-BOCS-scores from 22 to 9 after approx. 8.5 weeks, in patient 3 unfortunately no Y-BOCS history was available).

#### Possible autoimmune OCD

Possible organic OCD was observed in a female patient with suspected Hashimoto’s encephalopathy (with highly elevated thyroid antibodies—titer level was associated with clinical symptom severity, postinflammatory MRI lesions, anti-(peri)nuclear antibodies in the tissue test of serum and CSF, as well as blood-CSF-barrier dysfunction (Patient 6; cf. [35]), as well as in one patient with slight pleocytosis in CSF, non-specific white-matter changes, slightly elevated ANAs, and borderline myelin-binding in serum using tissue-based assays (Patient 7). The only patient with alterations in the FDG-PET (with a slight asymmetry of the metabolism of the association cortices with a low frontotemporal emphasis and a z-score of −2) was also considered as possible autoimmune OCD due to additional positive OCBs in CSF, strong antinuclear binding in serum and CSF using tissue-bases assays, severe rhabdomyolysis under risperidone augmentation, as well as an occipital gray matter reduction and CSF increase in automated MRI analyses (Patient 8).

#### Probable genetic OCD

Genetic exome analysis was initiated in two patients with suspected genetic syndromes. Both patients had a probable syndromal cause related to newly diagnosed genetic syndromes (Patient 4: duplication of chromosome 15q11-13 and Mayer–Rokitansky–Küster–Hauser syndrome; Patient 5: Baraitser–Winter syndrome) and brain involvement (Patient 4: hearing loss, reduced intelligence quotient (IQ), bilateral orbitofrontal/frontal gray matter decrease in the automated MRI analyses; Patient 5: hearing loss, reduced IQ levels, small parenchymal brain defect right temporal after cavernomectomy, and a volume loss in the parietal/occipital cortex, left orbitofrontal, and the right mesiotemporal cortex) [36].

#### Possible genetic OCD

Furthermore, two patients with pre-diagnosed chromosome disorders involving the X chromosome (Patient 9: Triple X syndrome; Patient 10: Turner syndrome) and morphometric MRI alterations (bilateral (Patient 9), left (Patient 10) decreases in orbitofrontal gray-matter volume) were classified as possible organic cases [37].

**Fig. 3:**
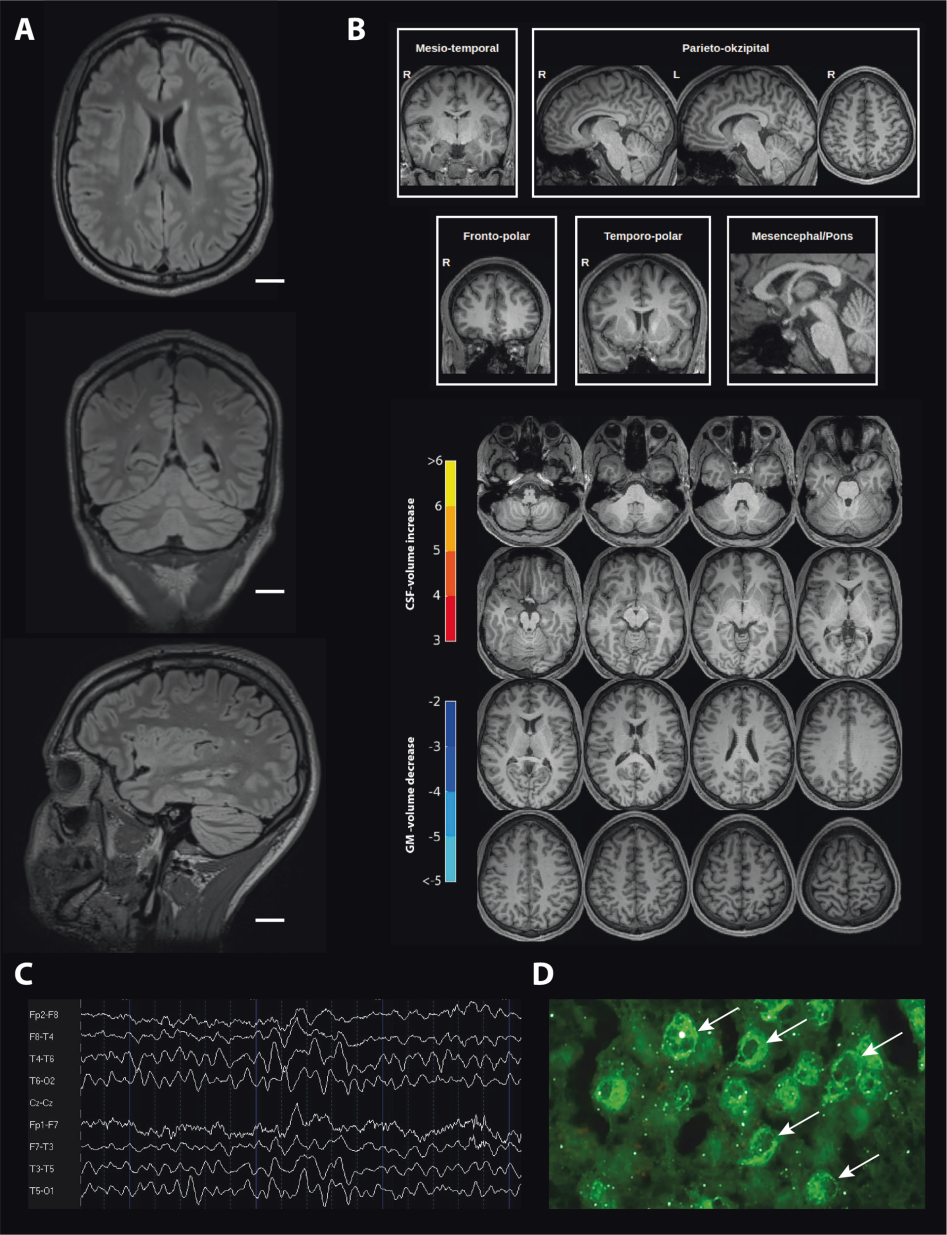
Exemplary diagnostic findings as may be found in some patients with OCD. **A** MRI with postinflammatory white matter lesions in FLAIR-sequences. **B** Automated Veobrain MRI analyses of the same patient shows normal findings without atrophic changes **C** EEG finding with intermittent generalized slowing. **D** Perinuclear staining (which were identified in serum and cerebrospinal fluid of some OCD patients) on unfixed murine brain slices (Prof. Prüß, Charité Berlin). FLAIR Fluid-attenuated inversion recovery, GM gray matter, FDG-PET fluorodeoxyglucose *-* positron emission tomography.

All organic cases are summarized in detail in Table 6 and exemplary findings are depicted in Fig. 3.

**Table 6:** Characteristics of probable and possible organic OCD patients.

Case	Sex	Age	OCD symptoms	Somatic comorbidity	Immunological changes	CSF	Tissue-based assays	MRI and FDG-PET	Changes in Veobrain analyses	EEG (IRDA rate)
*Probable organic OCD*										
Pat. (1)	F	Mid 20	Mixed obsessional thoughts and acts	None	Elevated CRP (+)	Pleocytosis (10 cells), increased IgG index and elevated lactate, positive OCB in CSF	Abs against cilia of granule cells in CSF (not in serum)	MRI: post-inflammatory lesion of left tapetum, pinealis cyst FDG-PET inconspicuous	Inconspicuous	Inconspicuous (0/min)
Pat. (2)	M	~60	Mixed obsessional thoughts and acts	Arterial hypertension	ANA 1:400 with anti-Jo1 and anti-mitochondrial M2 abs, elevated CRP and anti-TPO abs	Inconspicuous	Abs against neuropil in basal ganglia (and brain stem) in CSF and serum	MRI: bilateral small white matter lesions, paraventricular defect (left posterior horn) FDG-PET: inconspicuous	Bilateral orbitofrontal CSF-volume reduction on both sides	Inconspicuous (0/min)
Pat. (3)^a^	M	Mid 20	Mixed obsessional thoughts and acts, comorbid psychotic symptoms initially	Neuropsychiatric lupus	ANA 1:800 with anti-nucleosome specifity, anti-streptolysine and anti-streptococcal-DNAseB abs positive	Pleocytosis (14 cells), slightly increased total protein, positive OCB in CSF	Not investigated	MRI: multiple periventricular white matter lesions	Inconspicuous	Inconspicuous (0/min)
Pat. (4)	F	Mid 40	Mixed obsessional thoughts and acts	Duplication of chromosome 15q11-13, Mayer–Rokitansky–Küster–Hauser syndrome	None	Not investigated	Not investigated	MRI: inconspicuous	GM-Volume reduction orbitofrontal on both sides, frontal on both sides, parietal right	Inconspicuous (0.36/min)
Pat. (5)	M	Mid 20	Predominantly compulsive acts	Baraitser–Winter syndrome	Anti-streptolysine and anti-streptococcal-DNAseB abs positive	Not investigated	Not investigated	MRI: small parenchymal brain defect right temporal after cavernomectomy	GM-Volume reduction parietal on both sides, CSF-volume reduction orbitofrontal left, mesiotemporal right, occipital on both sides	Inconspicuous (1.53/min)
*Possible organic OCD*										
Pat. (6)	F	~40	Mixed obsessional thoughts and acts	Arterial hypertension	Elevated anti-TPO and TG abs	Slight BBB-dysfunction (AQ 8.1)	Anti-nuclear binding with a perinuclear pattern (stronger in CSF than serum)	MRI: bilateral postinflammatory white matter lesions	Inconspicuous	Inconspicuous (0.40/min)
Pat. (7)	M	~40	Predominantly obsessional thoughts	None	ANA 1:200, elevated anti-TPO and TG abs	Pleocytosis (6 cells), BBB-dysfunction (AQ 14.3, TP 927 mg/l)	Borderline anti-myelin-binding in serum (+)	MRI: non-specific WM lesions	Volume loss in the OFC on both sides and in the left parietal cortex	Intermitted generalized slowing (0.62/min)
Pat. (8)	M	End 40	Predominantly obsessional thoughts or ruminations	Rhabdomyolysis (CK max. 56870 U/l), latent hypothyroidism, arterial hypertension	Anti-streptolysine abs positive, elevated CH50	Positive OCB in CSF	Strong anti-nuclear binding with a perinuclear pattern in CSF and serum	MRI: inconspicuous FDG-PET slight asymmetry of association cortices metabolism (R < L) with a low frontotemporal emphasis (z-score −2)	GM-Volume reduction occipital right, CSF-volume increase occipital left and in cerebellum	Inconspicuous (0.11/min)
Pat. (9)	F	~20	Mixed obsessional thoughts and acts	Triple X syndrome	Elevated CRP (+)	Not investigated	Not investigated	MRI: small pinealis and pituitary pars intermedia cysts	GM-volume reduction orbitofrontal/frontal/ parietal/occipital on both sides, temporal right and central left	Intermitted generalized slowing (6.62/min)
Pat. (10)	F	Mid 20	Predominantly compulsive acts	Turner syndrome, Hashimoto’s thyroiditis	Elevated anti-TG abs	Not investigated	Not investigated	MRI: inconspicuous	GM-Volume reduction orbitofrontal left and mesiotemporal left	Inconspicuous (0.11/min)

*ANA* antinuclear antibodies, *AQ* albumin quotient, *BBB* blood–brain barrier, *CK* creatine kinase, *CRP* C-reactive protein, *CSF* cerebrospinal fluid, *DNA* deoxyribonucleic acid, *EEG* electroencephalogram, *F* female, *FDG-PET* fluorodeoxyglucose - positron emission tomography, *GM* gray matter, *IRDA* intermittent rhythmic delta activity, *L* left, *M* male, *MRI* Magnetic resonance imaging, *OCB* oligoclonal bands, *OCD* obsessive compulsive disorder, *R* right, *TG* thyroglobulin, *TP* total protein, *TPO* thyroid peroxidase.

^a^Neuropsychiatric lupus was diagnosed at an earlier stage.

## Discussion

The FDP-OCD screening detected probable or possible organic forms of OCD in 16%, as well as relevant diagnostic alterations in the overall patient sample.

### Probable and possible organic OCD cases

Ten patients were considered to have organic OCD subtypes, whereby five patients (8%) were considered to have probable OCD (three patients with autoimmune causes and two patients with new genetic syndromes), and five patients (8%) were considered to have possible organic OCD (three with autoimmune causes and two patients with genetic syndromes).

#### Autoimmune-mediated OCD

Detection of autoimmune OCD is of particular importance because causal immunotherapies are available for treatment. According to recent international consensus criteria, autoimmune OCD is confirmed by the detection of specific neuronal antibodies in CSF and/or a response to immunotherapy [16]. This was the case for two patients in the present cohort (3%). Both patients had abnormalities in the immunological laboratory examinations and in the MRI; therefore, a CSF analysis was added. The first patient showed a specific pattern against granule cells that could result in glutamatergic dysregulation. This patient showed full remission under high-dose glucocorticoid treatment [34]. The second patient had specific antibodies against basal ganglia in combination with a volume reduction in the OFC, which could have induced a dysbalance of the cortico–striato–thalamo–cortical loops. This patient also showed significant improvement under glucocorticoid treatment [20]. A third patient had neuropsychiatric lupus erythematosus with isolated psychiatric manifestations (besides OCS also briefly psychotic symptoms) and clear diagnostic abnormalities with anti-nucleosome specificity and inflammatory CSF signals (pleocytosis, OCBs). This patient also received immunosuppressive therapy and initially showed clear improvement. These three cases (5%) impressively demonstrate the importance of an advanced diagnostic work-up and the potential of immunotherapy in some patients with autoimmune OCD (whereby placebo effects clearly cannot be excluded). As possible autoimmune OCD was considered one patient who had several borderline findings like slight pleocytosis in CSF, non-specific white-matter changes in MRI, borderline myelin-binding, and ANA, while another patient had conspicuous FDG-PET with positive OCBs in CSF and strong antinuclear binding in tissue-based assay. In addition, one patient had questionable Hashimoto’s encephalopathy [35]. Due to the borderline findings, immunotherapy was not initiated in these three cases. Currently, in clinical practice, decisions for immunotherapy are complex scenarios made on a case-by-case basis, especially in possible cases. Further research is needed to clarify if specific immunological causes are present in such cases and whether these patients profit from immunosuppressive or immunomodulatory therapies.

#### Genetic syndromes

Furthermore, genetic syndromes were assessed as possible and probable organic OCD. The duplication of chromosome 15q11-13 and Mayer–Rokitansky–Küster–Hauser syndrome in one patient and the Baraitser–Winter syndrome in another [36] were considered to be likely related to their OCS. Both patients showed other central nervous changes such as hearing loss, reduced IQ level, and matching MRI pathologies. Two patients with chromosome disorders involving the X chromosome (Turner syndrome and triple X syndrome) were assessed as having possible organic OCD [37]. Interestingly, all these patients showed orbitofrontal volume reduction in an automated MRI evaluation, which could be associated with dysfunction in the cortico–striato–thalamo–cortical loops [9]. Such well-defined genetic cases with brain involvement may also provide novel insights into OCD pathophysiology.

### Diagnostic findings in the entire OCD patient group

#### Autoimmunity and antibodies

A high rate of endocrinological disease in 25% of cases was detected, with prominence of thyroid disorders with hypothyroidism in 13% and Hashimoto’s thyroiditis in 7%. Elevated anti-TPO in 19% and anti-TG antibodies in 13% were observed. Hereby elevated TPO antibodies were twice as common as in other reported control groups at 9.1% [38]. It has already been observed that OCD is more frequent in patients with Hashimoto’s thyroiditis [39] and other thyroid diseases [40], including papillary thyroid cancer [41]. The increased risk for Hashimoto’s thyroiditis in OCD was estimated to be 59% [42]. One patient was diagnosed with possible Hashimoto’s encephalopathy (cf. [35]). In such cases, therapeutic alternatives with immunotherapies might emerge [35].

However, other autoantibodies were also detected to an increased extent. Positive ANA findings were observed in 36% of the cases. In comparison, positive ANA findings of grades 1 to 4 have been found in only 16% of the general population in the United States of America [43]. However, only a few specific antibodies directed against extractable nuclear antigen (ENA) could be identified, with one patient having systemic lupus erythematosus with brain involvement. Prior data on ANA findings and OCS are limited to a few case presentations with suspected secondary OCD and positive ANAs [21, 44]. However, there is a known increased risk of any immune disease in OCD patients of 43%, with the highest risk of Sjögren’s syndrome at 94% [42]. Further research is needed to show whether there are specific ENA in OCD [33]. Well-characterized neuronal autoantibodies [45] could not be detected in the present cohort. In a subgroup of patients with suspected organic OCD, neuronal antibody binding using tissue-based assays on murine brain slices [29] was identified in two patients with a novel but specific pattern in CSF. This suggests that novel neuronal autoantibodies may play a role in a subgroup of patients with OCD. When considering organic causes of OCD, PANDAS is usually the first thing that comes to mind. Therefore, the investigation of streptococcal antibodies (anti-streptolysin and anti-streptococcal DNAseB) are of great interest in OCD. Elevated streptolysin antibodies were detected in 40% of patients in the cohort, which is more than in a healthy control group of Han Chinese (14%) [46]. Regarding the anti-streptococcal-DNAseB antibodies, the prevalence was comparable to the healthy adult Chinese population when applying the reference value of 480 IU/ml (6.4% in OCD patients of this cohort and 5.3% in healthy Chinese controls). Although streptococcal infections have been shown to increase the risk of OCD, it is unclear whether PANDAS or maybe unclassified milder forms are responsible [47]. In this cohort, none of the OCD patients with high anti-streptococcal antibody titers fulfilled the clinical criteria for PANDAS [48] in childhood and/or disease onset. Therefore, no therapeutic consequences followed from these findings.

#### Standard laboratory analysis and vitamin deficiencies

Increased serum calcium concentrations, which have previously been described in OCD [49], were noted. Interestingly, primary hyperparathyroidism with elevated calcium levels can present with OCS [50], and calcium-signaling pathways may play an important role in SSRI treatment response [51]. Elevated triglycerides were found in 25% of patients, which is significantly higher than in the general population (around 10%) [52]. In the literature to date, there are no known links between OCD severity and diet quality or nutrient intake [53]. In recent years, vitamin D has been discussed as a possible trigger and modifier of mental illness. Although most samples were collected in summer, 75% of OCD patients showed vitamin D deficiency at suboptimal levels. Reduced vitamin D levels have been described in children with OCD [54] or PANDAS [55], although more recent studies have found no significant differences in children with OCD [56]. In a recent meta-analysis of these studies, no association was found between OCD and vitamin D deficiency [57]. Nevertheless, vitamin D may have a disease-modifying effect on OCD [58]. Another vitamin, folic acid, was significantly decreased in 24% of the patients. In some studies, this was already reported for OCD [59]. Our patients with vitamin D and folic acid deficiencies were offered substitution therapy. A noteworthy reduction in vitamin B12 levels as reported in a meta-analysis [57] was not observed in this patient cohort.

#### MRI and EEG alterations

MRI showed abnormalities in 39% of patients, with 19% potentially relevant to OCD in the form of white-matter lesions due to post-inflammatory processes. While no atrophy was visually clearly apparent, automated analysis revealed either a decrease in gray-matter volume, an increase in CSF volume (indirect signs of discrete volume loss) or both in 45% of the examined MRIs. These findings do not necessarily indicate a loss of volume or atrophy, but may also be due to a neurodevelopmental process caused by e.g., genetic syndromes. Frequently, orbitofrontal or frontal areas in general were affected, which is consistent with alterations of the well-known cortico–striato–thalamo–cortical circuits in OCD [9]. The orbitofrontal cortex in particular showed increased functional/metabolic activity in functional MRI and PET studies, as well as a decreased density in postmortem studies in OCD [60–62]. However, parietal and occipital areas were also partially affected in the automated MRI analysis, which has also been previously reported for OCD and could possibly be associated with the impairment of the fronto–posterior tracts in OCD [9, 63]. Automated MRI approaches could have additional diagnostic value in the diagnostic work-up of patients with OCD. Evidence of epileptic activity was not found in the EEGs, and EEG slowing was relative rare (7%). No patient was thought to have an epileptic genesis, and signs for “paraepileptic changes”, that could potentially have therapeutic consequences [64], were also rare.

### Limitations

Due to the retrospective approach in a naturalistic setting, not all results were available for all patients and only cases with suspected organic OCD received additional diagnostics including lumbar punctures and FGD-PET. In addition, the neuropsychological screening could have been broader. Altogether, the patient group could have been larger and in the absence of a control group, only comparisons with reference values and published prevalence of abnormalities in the general population were possible. Finally, the classification into probable or possible organic OCD forms was based mainly on preliminary criteria [16].

## Conclusions

A relevant number of organic OCD forms (in 16%) were identified, which in 5% of all patients lead to successful treatment with immunotherapies. The frequent presence of autoantibodies such as anti-TPO or ANA further support the possible influences of autoimmune processes in OCD [16]. Further findings may well have modulatory effects on the course of the disease (e.g., substitution of folic acid deficiency). In addition, these findings might have a positive influence on the disease concept and self-image of affected patients and their relatives. The establishment of diagnostic regimens such as FDP-OCD should be evaluated in larger prospective and controlled studies. This could allow for a specific treatment of a small subgroup of patients with identified organic OCD reducing treatment resistance.

## Supplementary information

Supplementary Table

## Data Availability

All necessary data can be found in the article.
