# Supplementary material for: Deep clinical phenotyping of patients with obsessive-compulsive disorder: an approach towards detection of organic causes and first results
Source: Transl Psychiatry. 2023 Mar 7;13:83. doi: 10.1038/s41398-023-02368-8 (PMC9992508; doi:10.1038/s41398-023-02368-8)
Supplement: Supplementary file 1 — Supplementary Table [file 41398_2023_2368_MOESM1_ESM.docx]

**SUPPLEMENTARY MATERIALS**

| **ECG findings (*N*=60)** | |
| --- | --- |
| Alterations  No alterations | 10 (17%)  50 (88%) |
| Cardiac arrhythmias   - Sinus arrhythmia - Ventricular extrasystoles - Congenital paroxysomal reentrant tachycardia | 1 (2%)  1 (2%)  1 (2%) |
| Bundle branch block   - Left anterior hemiblock - Left bundle branch block - Incomplete right bundle branch block | 2 (4%)  1 (2%)  2 (4%) |
| Hypertrophy   - Left heart | 3 (5%) |
| Long-QT (>460 ms for women, >450 ms for men) | 4 (7%) |
| Others   - Notching | 1 (2%) |

**Supplementary Table S1: ECG findings.** Abbreviation: ECG = Electrocardiography.
